# A comprehensive electrooculography measurement system and protocols for screening myasthenia gravis

**DOI:** 10.1016/j.mex.2026.103810

**Published:** 2026-02-02

**Authors:** Tien Loc Le, Mark I. Boulos, Donna Yang, Sarah Berger, Mona Irannejad, César Morales Figueroa, Kevin E. Thorpe, Brian Murray, Sridhar Krishnan, Arun N.E. Sundaram, Hans Katzberg, Karthikeyan Umapathy

**Affiliations:** a Toronto Metropolitan University, Toronto, Ontario, Canada; b Hurvitz Brain Sciences Research Program, Sunnybrook Research Institute, Department of Medicine, Division of Neurology, University of Toronto, Sleep Laboratory, Sunnybrook Health Sciences Centre, Toronto, Ontario, Canada; c University Health Network – Toronto General Hospital, Toronto, Ontario, Canada; d Dalla Lana School of Public Health – University of Toronto, Toronto, Ontario, Canada

**Keywords:** Signal processing, Signal acquisition, Electrooculogram, Electrooculogram protocols, Myasthenia gravis

## Abstract

Myasthenia gravis (MG) is a neuromuscular disorder that can precipitate serious and fatal complications, especially when respiratory muscles are affected. Current electrodiagnostic methods, such as Single Fiber Electromyography (SFEMG) and Repetitive Nerve Stimulation (RNS), have notable limitations. While SFEMG is sensitive to neuromuscular abnormalities rather than being specific for MG, it is also a semi-invasive procedure that requires supervision by specialist clinicians and an expensive clinical setup. Conversely, RNS is highly specific for MG, but its sensitivity decreases in cases of low severity. Prior studies, including our previous work, have demonstrated that the manifestation of MG in affecting eye movements can be indirectly quantified using electrooculography (EOG) signals. This non-invasive method could develop into a widely used and easily deployed screening method, especially for early detection of MG using readily obtainable biomarkers. With this goal in mind, our research team developed data collection protocols using a standardized eye movement protocol that could be implemented in a standard clinical setting to acquire EOG data. In this article, we present the experimental setup, test protocols, sample outcomes, and preliminary analysis approaches. Common artifacts encountered, as well as technical and logistical challenges associated with such a setup, are also discussed.

1. An approach to non-invasively detect and quantify MG using eye movement signals.
2. Data collection protocols for acquiring eye movement signals designed for MG quantification
3. Novel eye movement-based biomarkers in quantifying MG.

## Graphical abstract

**Figure:**
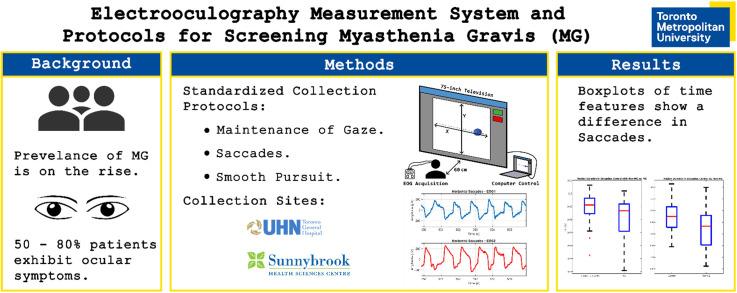

## Specifications table

**Table:** 

**Subject area**	Engineering
**More specific subject area**	Measurements and Signal Processing
**Name of your method**	EOG Measurement System and Procedure
**Name and reference of original method**	Journal Article: T. Liang, M. I. Boulos, B. J. Murray, S. Krishnan, H. Katzberg, and K. Umapathy, “Analysis of electrooculography signals for the detection of Myasthenia Gravis,” *Clinical Neurophysiology*, vol. 130, no. 11, pp. 2105–2113, Aug. 2019 Abstract: H. Katzberg et al., “Non-invasive Classification of Myasthenia Gravis and Other Ocular Disorders Using Electrooculogram Features (P1–11.008),” *Neurology*, vol. 104, no. 7_Supplement_1, Apr. 2025, doi: https://doi.org/10.1212/wnl.0000000000209072.
**Resource availability**	Compumedics Somte - https://www.compumedics.com.au/en/products/somte/

## Background

Myasthenia gravis (MG) is a neuromuscular disorder that affects the neuromuscular junctions in muscles, resulting in reduced muscle control. In severe cases, MG can be fatal, particularly when it affects the respiratory muscles. Recently, there has been a noted increase in the prevalence of MG [1], highlighting the urgent need for research to enable early detection before more severe conditions, such as generalized myasthenia gravis (gMG), develop. In 50–80 % of patients, ocular symptoms, including drooping eyelids (ptosis) and weakness of the extraocular muscles, have been observed as early signs before progressing to gMG [2]. Current standard clinical diagnostic methods include serological tests for specific antibodies, Single Fiber Electromyography (SFEMG), and Repetitive Nerve Stimulation (RNS). Although effective and routinely used in clinical practice, these methods have significant limitations. SFEMG is sensitive to neuromuscular issues, but it is invasive, requiring a fine needle, and is also expensive due to the need for specialized clinicians and cumbersome equipment [3]. In contrast, although RNS has higher specificity for MG compared to SFEMG, its sensitivity is lower, particularly in milder cases of MG [4]. Given these constraints and the fact that ocular symptoms often precede MG development, earlier studies, including our prior work [5], have attempted to quantify changes in eye movements to aid in the detection and severity assessment of MG [6].

Previous research on eye movement recording has utilized four primary modalities: electrooculography (EOG), magnetic search coil (MSC), infrared oculography (IROG), and video-oculography (VOG). MSC oculography was favoured in earlier studies due to its accuracy and reliability [7], which has given it a reputation as the gold standard for eye movement analysis. However, the influence of this method on participants’ eye movements has limited its applicability for clinical diagnosis, confining its usage primarily to experimental research. Subsequently, IROG and VOG have gained traction in recent studies, particularly regarding the effectiveness of VOG in assessing eye movements in patients with MG. In a recent VOG study, M. Ngoc et al. examined the gaze fixation in MG patients and observed that these individuals exhibited greater instability compared to healthy controls [8]. Similarly, Chae et al. assessed ocular muscle fatigue in MG patients by analyzing their saccadic movements using VOG, which revealed a diminished ability to sustain these movements in the affected individuals [9]. Both methods provide high accuracy, a broader range of recorded movements, and greater convenience [6,7]. Nevertheless, these studies faced limitations related to sample size, variations in task standardization, and feature extraction. Serological methods have also seen some advancement with the aid of image analysis. Specifically, serological methods with infrared spectroscopy have identified potential biomarkers in MG patients [10], but such studies can be sensitive to confounders such as the number and the handling of the collected blood samples. In contrast, oculography protocols take advantage of the established physiological connection between the eyes and MG [11]. As a result, these protocols may provide more straightforward and more interpretable feature sets.

EOG signals represent the simplest method for quantifying eye movements. Although EOG signals are susceptible to artifacts [7], their viability in the early detection of MG has been supported by several studies [6] and our previous research [5]. In our earlier work, EOG signals were collected from consenting participants during two sleep tests: the Multiple Sleep Latency Test (MSLT) and overnight polysomnography (PSG). We subsequently identified eye movements and extracted quantitative features using both conventional time-domain and advanced signal processing techniques. Classification based on these EOG-derived metrics demonstrated that even straightforward EOG signals could effectively reflect the neuromuscular abnormalities associated with MG, achieving reasonably high accuracy. Thus, further analysis and standardization of EOG-based features is warranted.

The current study builds on the foundation of our previous work by refining data collection methods to achieve a more comprehensive detection process while introducing standardization and repeatability to EOG analysis for MG screening. Our earlier work relied on EOG signals captured during sleep, which involved detecting spontaneous eye movements that could be infrequent and difficult to identify without manual verification. To address this potential problem, we proposed a standardized data collection process utilizing protocols designed to induce various types of eye movements. This approach not only facilitates automated detection but also streamlines the feature extraction process. Furthermore, to ensure that our methodology effectively differentiates MG from other conditions affecting the extraocular muscles, we have incorporated a non-MG cohort comprising patients with alternative neuromuscular disorders such as diplopia and palsy. By integrating structured movement protocols with a more diverse control group, we aim to establish a reproducible and non-invasive EOG-based framework for the early and specific detection of MG.

Our proposed methods directly benefit the clinical and medical communities involved in the diagnosis and longitudinal care of MG. Prominent organizations such as the Myasthenia Gravis Foundation of America (MGFA), the American Academy of Neurology (AAN), and the American Association of Neuromuscular & Electrodiagnostic Medicine (AANEM) play a crucial role in managing testing pathways for MG care. By implementing an objective, scalable screening approach, we can enhance early triage efforts, enabling healthcare providers to identify patients’ needs precisely. This approach will enable more tailored monitoring strategies and individualized therapy planning, ultimately improving patient outcomes and resource allocation in the management of MG.

From an engineering and technology perspective, the methodology provides significant value to biomedical engineers and device developers affiliated with organizations like the Biomedical Engineering Society (BMES) and the IEEE Engineering in Medicine and Biology Society (IEEE EMBS). It establishes a validated MG screening framework that defines clinical use cases and performance targets for signal processing and algorithm innovation. Medical device manufacturers and clinical engineering professionals in the Association for the Advancement of Medical Instrumentation (AAMI) can use this approach to develop non-invasive, workflow-compatible monitoring systems that integrate seamlessly with existing hospital infrastructure. Overall, this methodology bridges the gap between clinical needs and deployable technologies, enhancing early detection and disease management in MG.

The greatest long-term impact of the proposed screening methodology is on the general public, particularly patients with MG and related neuromuscular disorders, as emphasized by organizations like the Myasthenia Gravis Foundation of America and the Myasthenia Gravis Society of Canada. These communities often face challenges such as delayed diagnoses, misdiagnoses, and limited access to specialized testing. A non-invasive, accessible screening approach can expedite clinical referrals, reduce diagnostic journeys, and enhance quality of life through timely interventions.

A unique experimental setup has been created to facilitate the current study. Details of both the eye movement protocols, and the experimental procedures are discussed in detail in the following sections.

## Method details

EOG signals were measured using the Somté (Compumedics, Australia) [12] ambulatory polysomnography system and reviewed using its provided software. We employed a standard polysomnography setup to measure EOG, adhering to the technical specifications recommended by the American Academy of Sleep Medicine [13]. For the purpose of our study, other available Somté channels were not utilized. The rationale for using a standard polysomnography setup to measure EOG is to ensure consistency across the patients assessed in our study, as well as to facilitate the replication of our methodology by other investigators in a clinical setup.

### Clinical motivation for the protocols

EOG is a non-invasive, quick, and inexpensive method to quantify extraocular muscle function, the system most commonly affected early in MG. EOG signals capture the corneoretinal potential with surface electrodes [14]. By measuring this potential during standardized eye movement tasks, EOG can yield objective metrics including saccade amplitude and velocity, pursuit gain, fixation stability, and changes in these metrics over time that reflect fatigability. As previously mentioned, RNS and SFEMG tests are invasive as they require either electrical stimulation of peripheral nerves or insertion of fine-wire electrodes into muscle. In contrast, EOG relies solely on surface electrodes, so it avoids procedural pain, minimizes patient anxiety, and eliminates the risk of complications. The use of standardized visual stimuli, automated signal acquisition, and algorithm-based analysis also makes the test more operator-independent than RNS or SFEMG; thus, it reduces inter-operator variability and enables scalability across clinical settings.

Clinically, this approach can bridge the gap between symptom-driven examination and invasive electro-diagnostics. A structured EOG protocol can provide an early, reproducible signal of ocular MG involvement, help identify which patients require confirmatory testing with SFEMG or RNS, and support longitudinal monitoring of disease activity and treatment response. Improvement after immunotherapy, for example, can be tracked as normalization of saccade dynamics or reduced drift during sustained gaze. Its standardization enables comparability across sites, while the low burden promotes equitable access to diagnostic assessment. In this way, EOG offers quantifiable biomarkers of fatigability and ocular motor impairment that can augment current clinical pathways.

The experimental protocol was designed with neuro-ophthalmological principles in mind, targeting the extraocular muscle groups and ocular motor control systems most susceptible to MG-related fatigability. Maintenance-of-gaze tasks probe the ability to sustain eccentric fixation, revealing fatigable weakness of specific muscle pairs and uncovering subtle drift. Rapid saccadic movements assess both speed and consistency of ocular motor execution, which in MG may degrade with repetition due to impaired neuromuscular transmission. Smooth pursuit tasks engage cortical and brainstem pathways responsible for tracking moving targets, allowing us to detect breakdown in coordinated, slow eye movements under fatigue. By standardizing these paradigms across multiple gaze positions and movement types, the protocol maximizes sensitivity to the diverse patterns of ocular motor impairment seen in MG, while ensuring that results are physiologically interpretable in the context of known neuro-ophthalmic mechanisms.

As MG affects the neuromuscular junction and causes double vision, we are selecting patients with double vision for our non-MG cohort. Our focus includes individuals experiencing double vision due to other neurological causes, such as brainstem disorders, cranial nerve abnormalities, and myopathies. While these conditions exhibit clinical similarities, they should have distinct EOG features that will enable us to differentiate them from MG. Additionally, we are examining patients with double vision resulting from other etiologies, including minor strokes and genetic factors. Our methodology aims to effectively differentiate double vision caused by MG from that caused by alternative conditions.

### Measurement setup

**Fig. 1:**
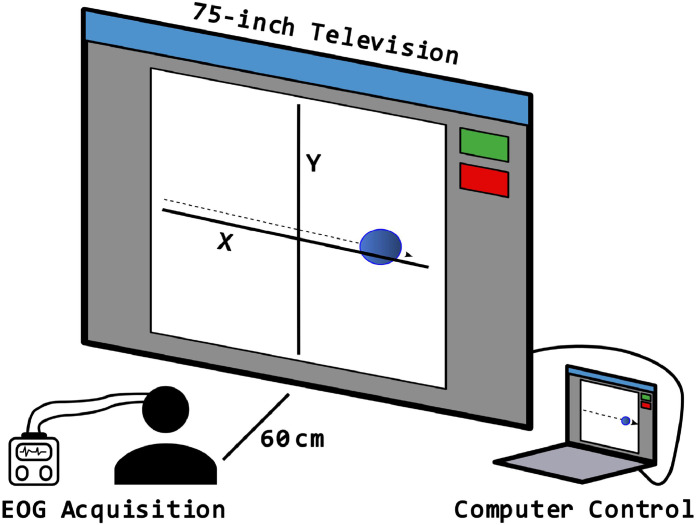
Measurement setup – Participant seated 60 cm from TV; screen dimensions *X* = *Y* = 91 cm; EOG acquired using Compumedics system (2 channels).

**Fig. 2:**
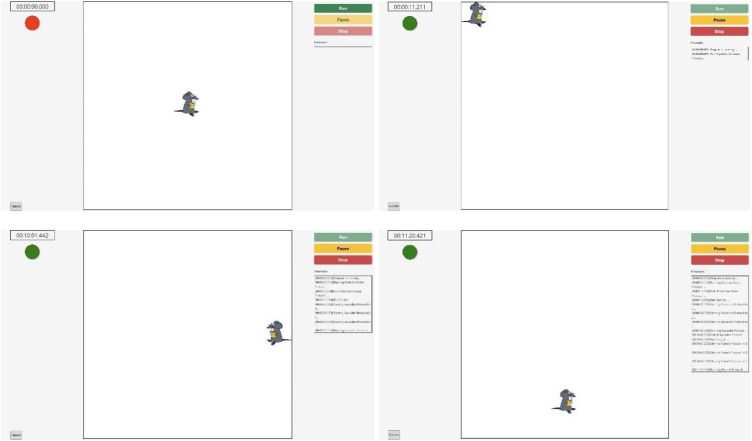
Measurement setup: Graphical user interface developed to perform the measurements. The red circle indicates the program is stopped, while the green circle indicates the program is running.

To collect EOG data from participants in a standardized setup, the authors designed the configuration as shown in Fig. 1. This setup included a Compumedics EOG acquisition system (Model Number: 7023–0005–01), a 75-inch Television, and a laptop. Custom software was developed to control the video displayed on the television. The computer presented a video of cartoon mouse stimulus (Fig. 2) that moved around the television screen according to predefined trajectories. Participants were instructed to visually follow the mouse within the set time limits of the protocol. Data was collected at two clinical sites: Toronto General Hospital and Sunnybrook Health Sciences. The study was approved by the ethics boards of both hospitals and the Toronto Metropolitan University (TMU). Data were analyzed and quantified afterwards at TMU. Patient recruitment was conducted in accordance with the approved ethics protocols. On the testing day, patients/subjects were briefed about the study and escorted to the experimental setup room. Once the registration process was completed, the patients (and normal controls for comparative study) were seated in a chair, approximately 60 cm in front of the television. The gold-plated EOG electrodes were affixed as per the EOG acquisition protocol manual to acquire both horizontal and vertical eye movements. Participants were instructed to respond to the stimulus only with their eyes while refraining from moving their heads.

### Data collection

1) EOG Recording

**Fig. 3:**
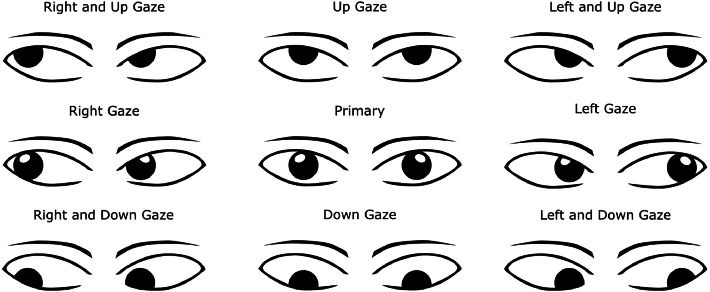
Eye positions for protocol 1 – Maintenance of Gaze.
• Protocol 2 – Saccades (4 min): Four 30-second periods of back-and-forth rapid eye movements from the primary position to the cardinal extremes (up, down, left, right), each followed by a 30-second rest.
• Protocol 3 – Smooth Pursuit (4.5 min): Vertical then horizontal tracking for 2 min each, separated by a 30-second rest.

All of the prospective study population (i.e. MG patients [from TGH and SBHSC], Non-MG [from TGH and SBHSC], and Controls [from TGH and SBHSC]) completed a single 30-minute seated EOG session. The recording sequence involved the participants performing uncontrolled eye movements, followed by structured eye movements. EOG recordings of uncontrolled eye movements were collected for 10 min, with eyes closed for 5 min and eyes opened for 5 min, to compare similar modalities of eye movements under two basic paradigms (eye closed/open status). Following the 10 min of uncontrolled eye movements (which should not cause fatigue for subsequent tests), EOG recordings of structured eye movements in the four primary extremes of gaze were recorded (total time 16 min). The structured eye movements are induced through the following three protocols:

• Protocol 1 – Maintenance of Gaze (7.2 min): Eight extreme positions (up, down, left, right, and four diagonal) held for 30 s each, interleaved with 10-second rest at the primary position, and ended with a 120-second rest. The eight positions for this protocol are illustrated in Fig. 3. These cardinal positions of gaze should subject all six extraocular muscles in both eyes to fatigability.

The head was stabilized during all tasks. Performance indicators for EOG were collected after completion of testing (i.e. duration of test, patient-reported pain and discomfort on a Likert scale (0–10), and self-reported patient preference compared to routine MG diagnostic approaches).

2) Ptosis Evaluation

In addition to the structured EOG recording protocols, complementary clinical assessments were performed to capture ocular fatigability and benchmark the proposed methodology against established diagnostic tools. Ptosis was assessed as an established clinical marker of ocular muscle weakness. The ‘vertical fissure height’ (distance between the eyelids in mm) (VFH) and ‘marginal reflex distance’ (distance between the corneal light reflex and the upper eyelid in mm) (MRD) were measured on both sides with a caliper. Following a sustained-up gaze for 1 min, both VFH and MRD were repeated to check for fatigable weakness in the levator palpebrae superioris muscle. An initial analysis was performed with 20 participants from the Controls, 20 patients from the MG, and 11 recruits from the NMG group. The statistic for each group is tabulated in Table 1.

3) Clinical Electrophysiological Testing

**Table 1:** Summary of the recorded VFH and MRD for ptosis evaluation in the 3 Groups.

	Controls ($n_{1}=20$)	MG ($n_{2}=20$)	NMG ($n_{2}=11$)
**Baseline VFH Left (mm)**	9.55 ± 0.38	8.15 ± 0.47	8.30 ± 0.91
**After 1min VFH Left (mm)**	9.47 ± 0.35	7.70 ± 0.52	7.20 ± 0.93
**Baseline VFH Right (mm)**	9.55 ± 0.50	8.50 ± 0.38	8.60 ± 0.87
**After 1min VFH Right (mm)**	9.63 ± 0.47	8.50 ± 0.36	7.70 ± 0.80
**Baseline MRD Left (mm)**	4.25 ± 0.38	3.6 ± 0.30	4.10 ± 0.67
**After 1min MRD Left (mm)**	3.84 ± 0.28	3.20 ± 0.44	3.00 ± 0.56
**Baseline MRD Right (mm)**	4.70 ± 0.83	3.80 ± 0.21	4.30 ± 0.60
**After 1 MRD Right (mm)**	3.74 ± 0.25	3.80 ± 0.21	3.30 ± 0.47

We also conducted standard clinical electrophysiological tests, including RNS and SFEMG, on the MG group to evaluate how our proposed protocols compare against current diagnostic tools. In the RNS test, facial nerve on the more involved side of symptoms was stimulated with surface electrodes placed over the frontalis and nasalis muscles. Action potentials were recorded during a one-minute maximum effort exercise to measure the decrement in area and amplitude from 1st to 4th responses. Similarly, SFEMG tests were performed on the frontalis of the most affected side of ocular symptoms, focusing on evaluating the mean jitter, along with percentage of muscle pairs behaving abnormally and exhibiting blockages.

At this stage, 13 of the 20 MG patients in the initial analysis have completed both tests, while the remaining patients are still pending. Among the 13 patients who completed the tests, the average RNS baseline value was 5.63 %. Following 1 min of maximum effort, the average RNS value decreased to 4.82 %. However, after resting for 1 min, the average RNS value returned to a level similar to the baseline, measuring at 5.57 %. It is important to note that a decrement of more than 10 % between the 1st and 4th responses is considered abnormal [15]. Additionally, this group of MG patients exhibited a mean jitter of 86.76 µs, with an average of 32.13 % of muscle pairs behaving abnormally and 27.22 % of muscle pairs showing blockages. In contrast, a healthy individual typically has a normal mean jitter of approximately 20 µs, and the upper limit for muscle pairs with issues is 30 % [16]. The performance metrics, as stated above, were also captured during these tests (i.e. test duration, pain and discomfort on a Likert scale (0–10), patient preference compared to the EOG test).

### Sample signal plots

**Fig. 4:**
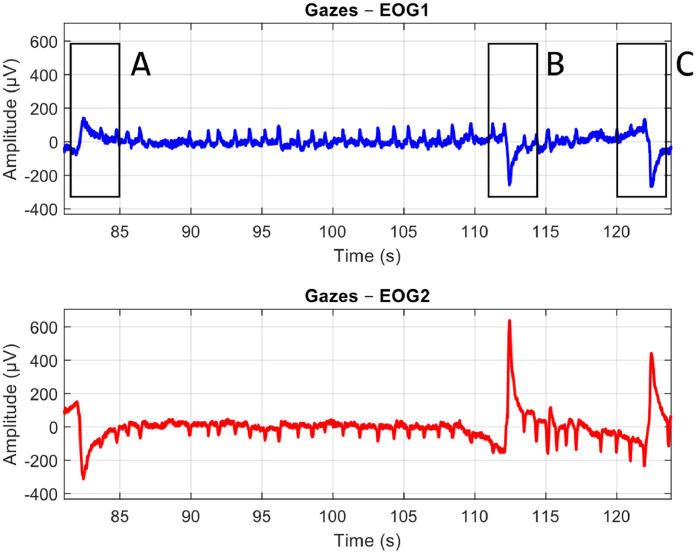
A Sample Plot of Raw EOG Signals with Artifacts and Noise for Maintenance of Gaze. EOG Channel 1 (Blue) and EOG Channel 2 (Red). Anti-phasic movements can be observed at marked A, B, and C locations to indicate participants following the target.

**Fig. 5:**
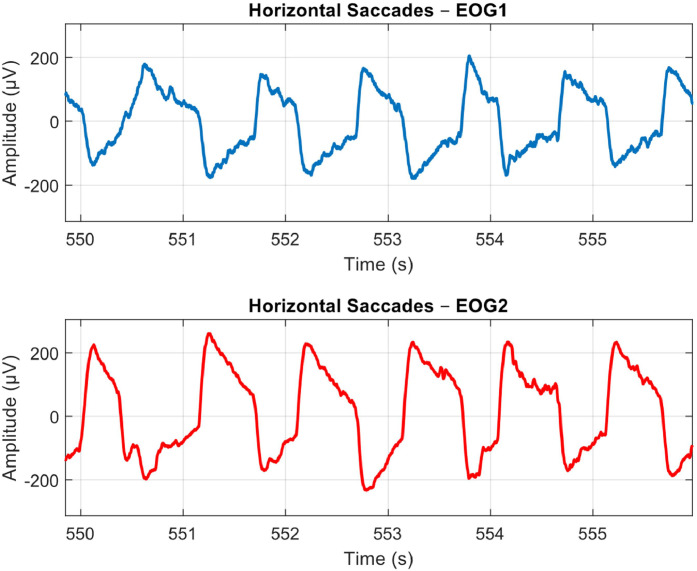
A Sample Plot of Raw EOG Signals with Artifacts and Noise for Horizontal Saccades. EOG Channel 1 (Blue) and EOG Channel 2 (Red). Anti-phasic movements are recorded as participants move their eyes from the center to the side and back over 30 s.

**Fig. 6:**
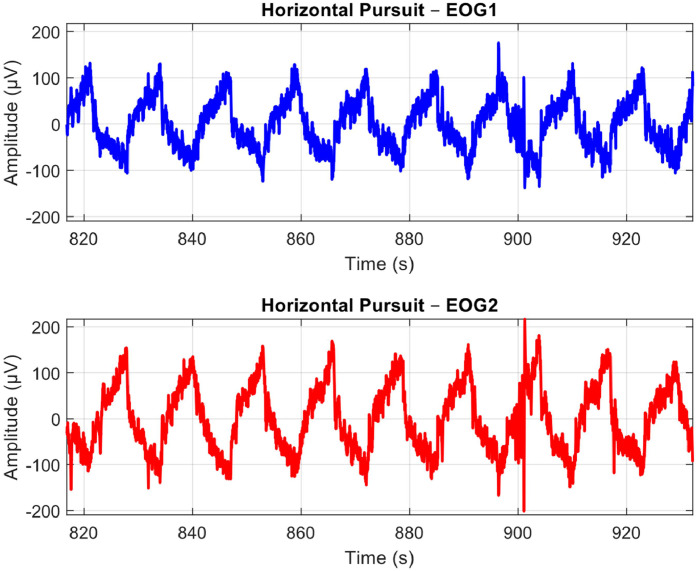
A Sample Plot of Raw EOG Signals with Artifacts and Noise for Smooth Pursuit. EOG Channel 1 (Blue) and EOG Channel 2 (Red). Anti-phasic slow drifts are recorded as the participants follow the target from one side to the opposite for 120 s.

The EOG signals from the two channels are derived after subtracting their respective reference signals. The signals are then filtered to remove noise and unwanted artifacts that fall outside the frequency range of interest for the eye movements. Figs. 4, 5, and 6 demonstrate the raw EOG signals for Maintenance of Gaze, Saccades, and Smooth Pursuit, respectively.

In the Maintenance of Gaze protocol, participants begin by keeping their eyes at the primary position. Following the projected target on the TV, they move their eyes to one of the eight extreme positions, and this initial movement is marked as A in Fig. 4. After maintaining their gaze for 30 s, they return their gaze to the primary position for a brief rest, marked by movement B in Fig. 4. This process is then repeated, with movement C in Fig. 4 marking the start of the next gaze cycle.

The Saccades protocol is conducted 2 min following the first protocol, giving participants sufficient time to recover from ocular muscle fatigue. As depicted in Fig. 5, participants rapidly direct their gaze back and forth between the primary position and one of the four extreme positions, creating swift and consecutive anti-phasic movements.

After a rest period at the end of the Saccades protocol, participants perform the Smooth Pursuit task, which is illustrated in Fig. 6. In contrast to the preceding protocols characterized by rapid and sharp anti-phasic movements, participants exhibit slower anti-phasic movements while tracking the target displayed on the TV from one extreme position to another.

In addition to the patterns observed for each protocol, the raw EOG signals captured in Figs. 4–6 contain some noise and artifacts. For instance, Fig. 4 shows several small movements that resemble eye movements being recorded. Thus, in the next section, we will present some of the major sources of noise and artifacts, as well as how these were addressed via advanced signal pre-processing approaches.

### Artifacts and noise

On preliminary analysis of the signals extracted from the three previously explained protocols, we observed a significant influence due to involuntary blinks by the patient, absolute signal amplitude variations, and incomplete or erroneous eye movements, either due to pathological conditions or when subjects lose focus on the moving target.

#### Blinks

These unintentional blinks appear as sharp peaks that override the actual eye movement signals and can introduce false triggers, especially when automating the detection of eye movements and feature extraction. A sample plot (Fig. 7a) shows the blinks (via black arrows) overriding the eye movement signal during a horizontal eye saccade experiment. By applying suitable filtering and detrending techniques, this is removed, and the signal, free from blinks, is shown in Fig. 7b. As blinks were extremely fast movements, usually observed as spikes in the signals, a bandpass filter of 0.1–35 Hz was applied to the signals to reduce the intensity of sudden spikes.

**Fig. 7a:**
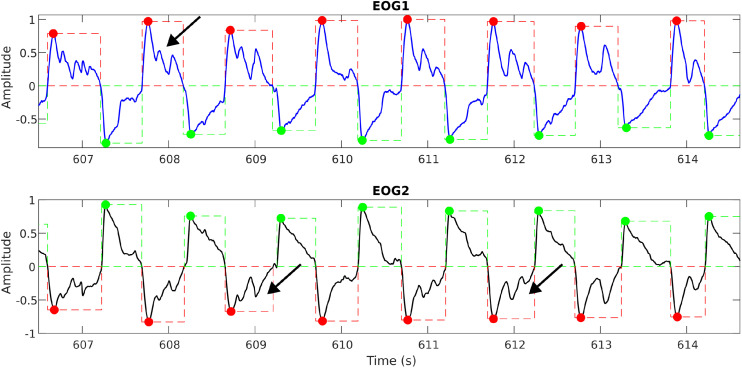
A sample plot showing the blinks (shown in black arrows) overriding the eye movement signal (Horizontal Saccade Protocol).

**Fig. 7b:**
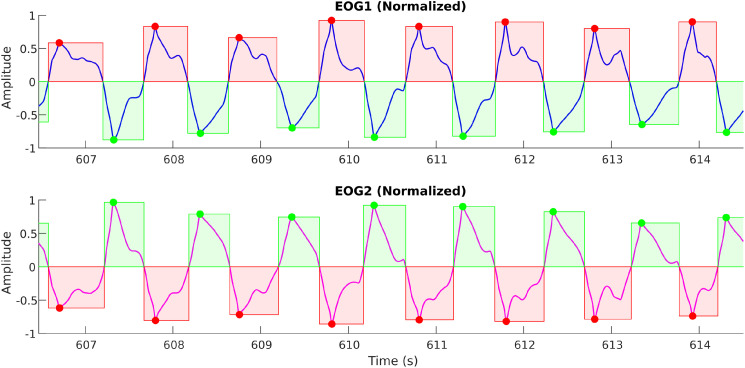
A sample plot showing the blinks removed eye movement signal. The green and red boxes show the detection of genuine eye movements.

The bandpass filter was followed by a smoothing process based on a locally weighted linear regression model (LOWESS) [17]. For each target point x[n] with n=0,1,2,…,N​, we fit a first‐degree polynomial by weighted linear least squares using data in the neighbourhood of [n]​.

The weights for each x[n] were computed with the following formula:

$$w_{i,\;n}={\left(1-{\left|\frac{x_{i}-x\left[n\right]}{h}\right|}^{3}\right)}^{3}$$

where $x_{i}$ (i=0,1,2,…,N) are the nearest neighbours of x[n] as defined by the desired smoothing distance h from x[n] [17].

#### Amplitude normalization

We have also noticed that due to variations in electrode contacts and inter-patient variabilities, many times the signal amplitude recorded on the two channels is not the same. When signal amplitudes are smaller than a set threshold or when the signals are affected by outlier artifacts, this affects the pre-processing, which in turn makes the automation of eye movement detection difficult. To avoid such cases, we need to adjust our amplitude normalization in such a way that it should not be biased by spurious outlier artifacts.

This is achieved by setting the normalization based on outlier detection so that the signal information is not lost. An example of this amplitude normalization is shown in Fig. 8a, Fig. 8b. As illustrated in Fig. 8a, when the EOG2 channel is not properly normalized to ignore outliers, it changes the perception of the actual signal amplitude; thus, it may lead to the erroneous discarding of genuine eye movements.

**Fig. 8a:**
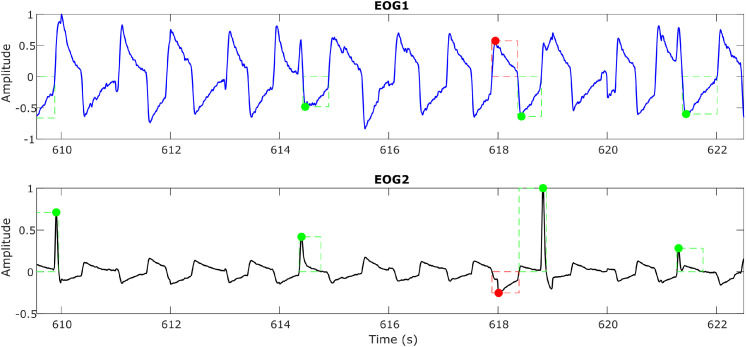
An example horizontal saccade signal demonstrating the effect of outliers on the amplitude normalization.

**Fig. 8b:**
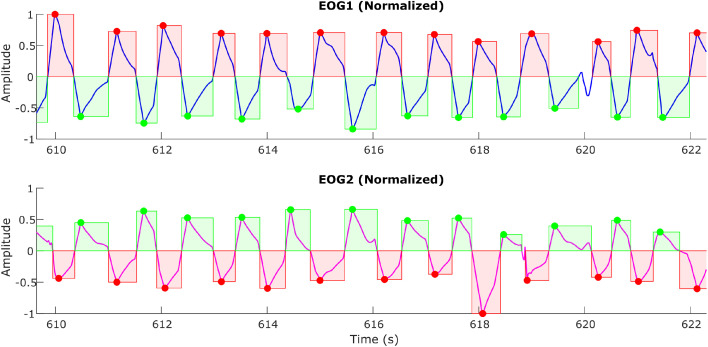
The above figure demonstrates the proper amplitude normalization after detecting and eliminating the outliers.

Amplitude normalization of each signal segment was conditioned on the presence of outliers determined by the Grubbs test [18,19]. Specifically, in segments with outliers, the segment is divided by the largest non-outlier value, ensuring that the remaining data points can be scaled and enhanced for the current procedures. Otherwise, the segment would be normalized by the maximum absolute amplitude. Outlier detection used the iterative Grubbs procedure, which computes a G statistic at each step [20].

$$G=\text{max}\frac{\left|x\left[n\right]-\bar{x}\right|}{s}$$

where s is the standard deviation and $\bar{x}$ is the mean of the segment. If the G value exceeds a relevant criterion, typically the critical value, the corresponding data point is considered an outlier and removed. The testing procedure repeats with N−1 data points until no more outliers are found. This approach ensures that normalization is robust to extreme values while preserving the dynamic range of non-outlying data.

#### Eye movement width

As explained earlier, subjects are expected to move their eyes in response to the mouse figurine projected on the screen. However, either due to pathological conditions or other distractions/difficulties, when the subject skips the periodic motion and tries to restart from the next cycle or catches up, this affects the integrity of the eye movements. Specifically, this affects the width (or span) of the eye movements in comparison to the average span for specific protocols. When this occurs due to a pathological condition, this will be one of the strong markers separating the pathological group from the normal controls; however, it can also happen due to non-pathological reasons. So, based on the eye movement tolerances that are possible physiological (with the inputs from the Clinical Experts), a limit was set to the eye movement width in order to separate the genuine eye movements (from MG, NC, and NMG groups) following the protocol from an erroneous artifact. This is demonstrated in Fig. 9a, Fig. 9b.

**Fig. 9a:**
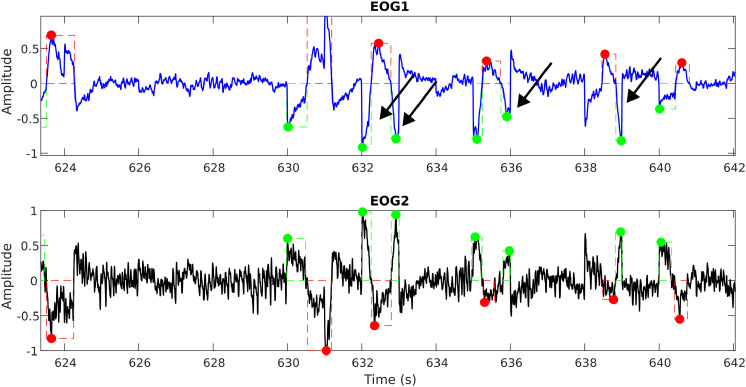
An example unprocessed horizontal saccade signal showing the erroneous detection of eye movements (at the right side of the plot) indicated by red and green boxes. Black arrows mark movements that resemble genuine eye movements.

**Fig. 9b:**
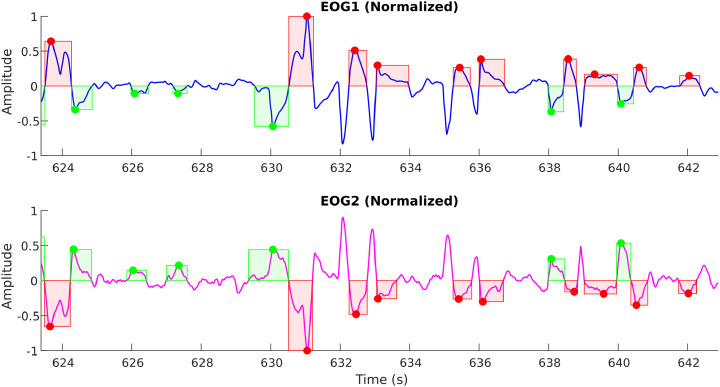
An example processed horizontal saccade signal with a width limit showing the detection of genuine eye movements (compare at the right side of the above plot with Fig. 9a), indicated by red and green boxes.

**Fig. 10:**
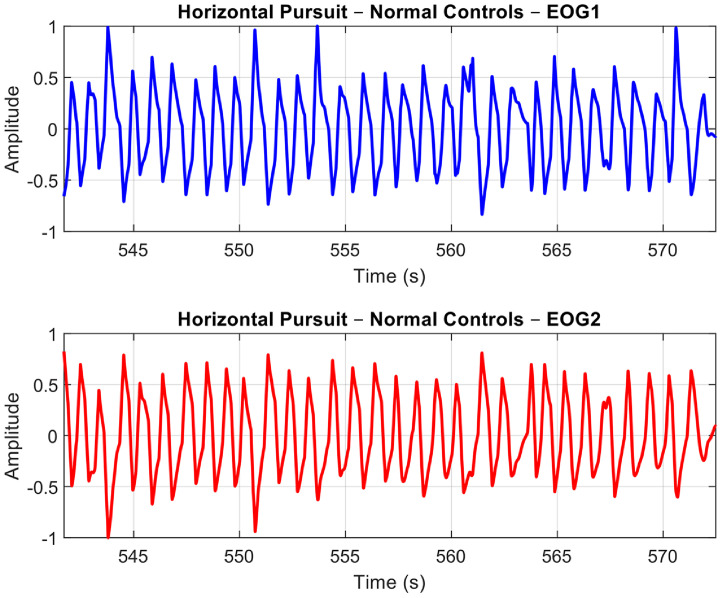
A Sample Normal Control Signal Plot. EOG Channel 1 (Blue) and EOG Channel 2 (Red) for Horizontal Saccade Protocol. An anti-phase characteristic of eye movement is observable in the plots.

**Fig. 11:**
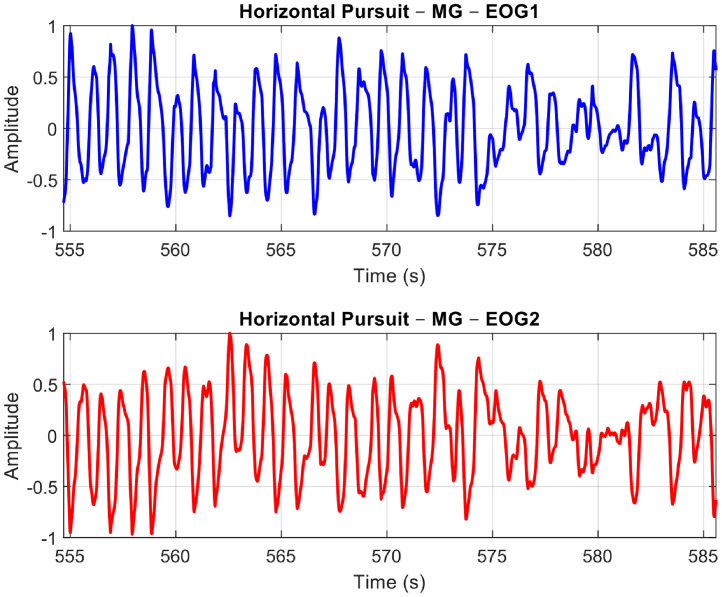
A Sample MG Signal Plot. EOG Channel 1 (Blue) and EOG Channel 2 (Red) for Horizontal Saccade Protocol. An anti-phase characteristic of eye movement can be observed in the plots.

**Fig. 12:**
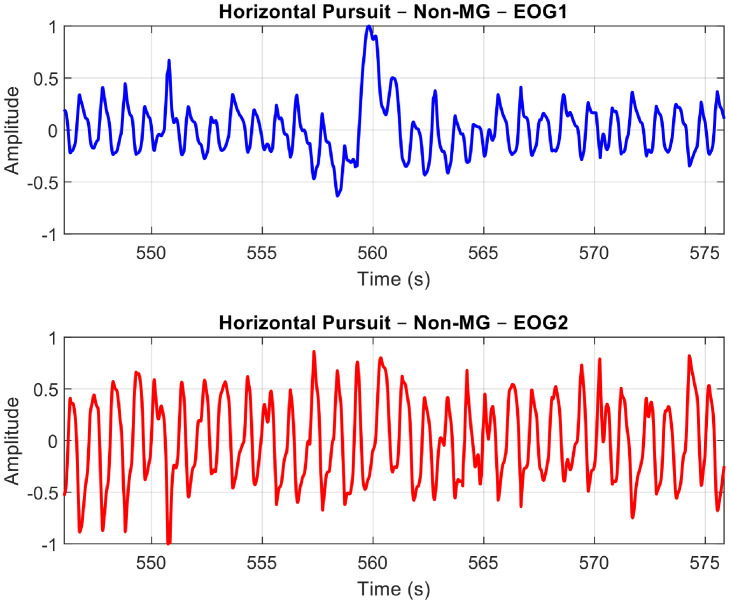
A Sample Non-MG (but other ocular disorder) Signal Plot. EOG Channel 1 (Blue) and EOG Channel 2 (Red) for Horizontal Saccade Protocol. An anti-phase characteristic of eye movement can be observed in the plots.

For the purpose of this paper, we will demonstrate and explain the signal pre-processing methods using our analysis with the Horizontal Saccades protocol. The following sample plots present the EOG signals obtained after pre-processing for the three groups: Normal Controls (see Fig. 10), MG (refer to Fig. 11), and Non-MG (as shown in Fig. 12), respectively.

Observing the three plots in Figs. 10–12, it is evident that the signal shape corresponding to a predefined eye movement as per the protocol (i.e. in this case, left saccades) is different. While the Normal Control signal shows greater repeatability of the eye movement, both MG and Non-MG signals exhibit differences in their eye movement span (i.e. width and amplitude) and regularity. To quantify these variations effectively, it is essential to isolate genuine eye movements from the EOG signals, and extract features from them. The subsequent section will outline our algorithm for detecting eye movements, along with the extraction of time-based features.

### Eye movement detection and time domain features

Eye movements were identified from the pre-processed signal $\tilde{x}\left[n\right]$ using a multi-step algorithm. First, on channel 1, candidate movement peaks were located as local extrema $p_{j}$. If the relative amplitude of the peak $\tilde{x}\left[p_{j}\right]$ is greater or equal the detection threshold T of the maximum amplitude in $\tilde{x}\left[n\right]$, $p_{j}$ is potentially a valid movement peak.

$$\tilde{x}\left[p_{j}\right]\geq T\times \;\text{max}\left(\tilde{x}\left[n\right]\right)$$

To distinguish genuine eye movements from noise, each peak in channel 1 was required to have a corresponding, antiphase deflection of comparable duration on channel 2. Finally, for each validated peak, precise onset $t_{start}$ and offset times $t_{end}$ were obtained by finding the nearest zero-crossings immediately before and after the peak. This procedure yields robust estimates of movement timing and amplitude, which are essential for subsequent feature extraction.

From each detected event, we extracted five temporal features: rise time $R_{t}$, rise rate $R_{r}$, fall time $F_{t}$, fall rate $F_{t}$, and width of the movement $W_{t}$. The time-based features measure the time difference between the onset/offset and the peak of the movement $P_{t}$, while the rate-derived features divide the peak amplitude by the time to obtain a slope of the upward or downward deflection. The width of the movement reflects the overall temporal extent of the movement. Additionally, the statistics of the time features, such as mean, median, and standard deviation, were calculated. Together, these features characterize the dynamics of each ocular event for subsequent analyses. The equations for those features are as follows:

• Rise time:
  $$R_{t}=P_{t}-t_{start}$$
• Rise rate:
  $$R_{r}=\frac{P_{t}}{P_{t}-t_{start}}=\frac{P_{t}}{R_{t}}$$
• Fall time:
  $$F_{t}=t_{end}-P_{t}$$
• Fall rate:
  $$F_{r}=\frac{P_{t}}{t_{end}-P_{t}}=\frac{P_{t}}{F_{t}}$$
• Width:
  $$W_{t}=t_{start}-t_{end}$$

**Fig. 13:**
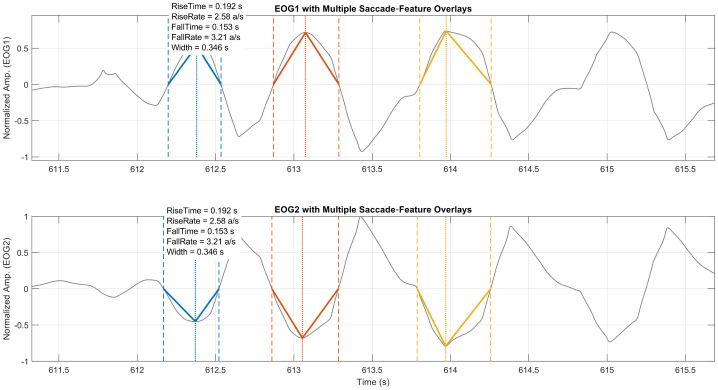
A Sample of Detected Eye Movements and Their Extracted Time Domain Features.

This signal processing procedure can be applied to the other protocols as necessary. By adopting a procedure, we can systematically evaluate each protocol. At the same time, each protocol can be examined separately so that different aspects of ocular motor control can be observed. In doing so, we can maximize the potential to reveal both shared and task-specific markers of MG-related impairment (Fig. 13).

## Method validation

Since this is a methods-focused paper, for the purpose of method validation, we will be highlighting our recently published results via an abstract [21]. In that abstract, we presented initial findings from our study involving 51 participants. The number for each group is 20 controls, 11 non-MG, and 20 MG, as determined by a physician’s clinical assessment. Each participant followed the proposed protocols as outlined previously. Our analysis focused on horizontal saccades, from which we extracted the aforementioned time features from the corresponding EOG signals. We evaluated the performance of each signal feature to binarily separate MG patients from the combined group of controls and non-MG patients (case A), and then a subclassification of controls and non-MG patients was performed (case B). The statistical significance of the group separations was determined using a simple Student’s *t*-test. Our results demonstrated a difference in one of the time features which is the median duration of the saccadic movements (MDS) per subject. For case A, we observed that the range of horizontal eye movements in MG patients was more restricted, leading to narrower movement widths than those in the controls and the non-MG group. Likewise, we found the non-MG group to exhibit a relative reduction in horizontal eye movements compared to the controls. We confirmed statistical significance in both cases, with a p-value ≤ 0.05. While further analysis is warranted, it is beyond the scope of this protocol paper. Fig. 14a, Fig. 14b illustrate the box plots summarizing the results presented in the abstract. Furthermore, these initial findings support the protocol’s design rationale, illustrating how saccade-based paradigms can capture ocular symptoms in MG patients.

**Fig. 14a:**
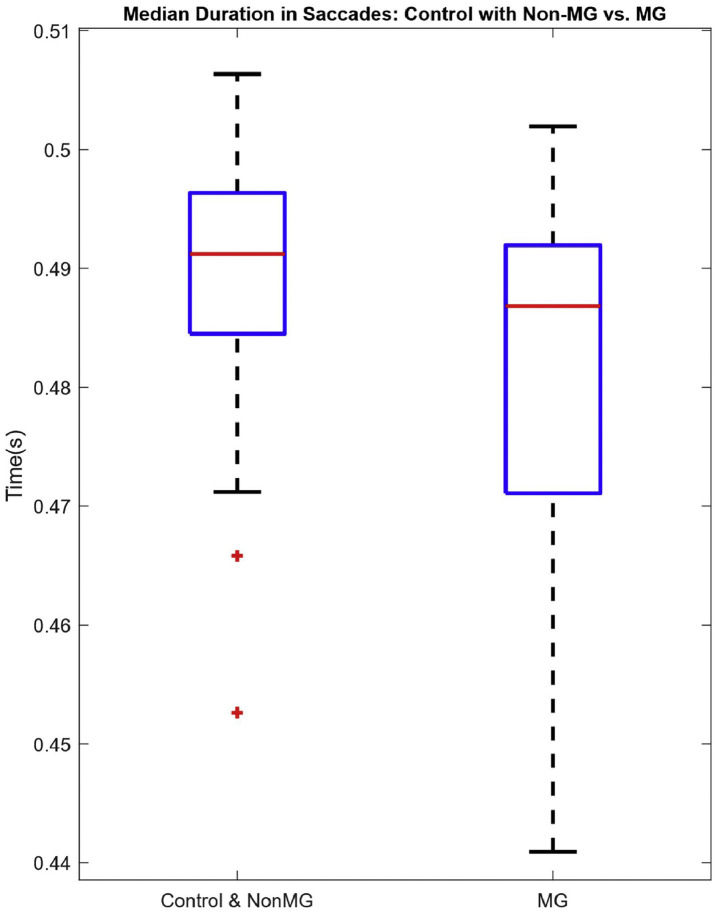
MDS per subject – Case A.

**Fig. 14b:**
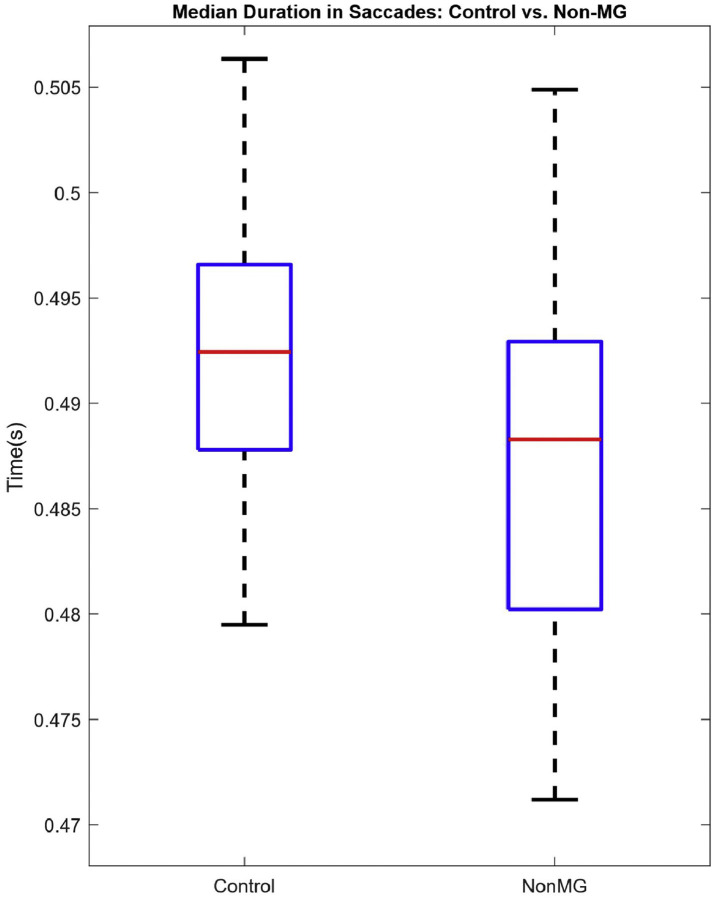
MDS per subject – Case B.

The sensitivity and specificity of the proposed method using the structured eye movement experiments is yet to be established. Nevertheless, our approach addresses several limitations of SFEMG and RNS concerning these metrics. Furthermore, the values obtained with SFEMG and RNS depend on the muscles and nerves selected for analysis. For ocular MG, the sensitivity of RNS fluctuates between 40 % and 60 % when examining facial nerve and muscle pairs. In comparison, SFEMG demonstrates a higher sensitivity, ranging from 90 % to 95 % for mild to moderate cases of MG. When it comes to specificity, RNS generally exhibits superior performance, achieving rates of 80 % to 90 % when a true physiological decrement of at least 10 % is detected. SFEMG, however, has notoriously lower specificity, falling between 60 % and 80 %, although this can be improved the more abnormal the SFEMG result is.

While the two traditional diagnostic methods serve as important standards for determining MG cases, our proposed methods are not meant to replace these two tools, but rather to enhance them. As non-invasive tools, they offer easier deployment, facilitating more accessible and timely screening. This accessibility can lead to prompt treatment for MG, thereby enhancing quality of life and helping to avert severe progressions of the condition.

This work presents comprehensive measurement system and data collection protocols for non-invasive EOG-based assessment of MG. By integrating controlled visual stimuli, structured eye movement protocols, and systematic signal processing frameworks, the approach provides reproducible and quantitative markers of ocular motor impairment in MG patients. The approach offers several advantages over conventional diagnostic methods, including reduced invasiveness and lowered burden on patients. With further validation, including these protocols, we could complement existing electrodiagnostic tests, support early screening, and enable prompt treatment of MG. Additionally, future work will involve developing advanced signal processing procedures to extract multi-protocol features, and biomarkers that can help detecting and quantifying the severity of MG.

## Limitations

Although the proposed protocols demonstrate feasibility, their limitations should be considered. EOG signals are inherently susceptible to artifacts such as blinks and participants’ inconsistent adherence to visual stimuli. While our signal processing pipeline addresses these challenges, complete artifacts elimination is not entirely possible; thus, it may affect signal interpretation. Besides, as the proposed protocols and the assessment methodology are new, they may not be all-inclusive. Additional multi-site validation and further studies may be required to standardize EOG reference values for clinical practice, especially in the diagnosis of MG.

## Ethics statements

All procedures were conducted in accordance with relevant laws and institutional guidelines and received approval from the appropriate institutional committee(s). The privacy rights of human subjects were respected, and written informed consent was obtained for the publication of anonymized patient information in this article.

## Related research article

None.

## CRediT author statement

Tien Loc Le – Software, Validation, Formal analysis, Writing – Original draft preparation, Visualization. Mark I. Boulos – Supervision, Resources, Conceptualization, Methodology, Writing – Review and Editing. Donna Yang – Data curation, Investigation. Sarah Berger – Data curation, Investigation. Mona Irannejad – Data curation, Investigation. César Morales Figueroa – Data curation, Resources, Investigation. Kevin E. Thorpe – Resources, Conceptualization, Methodology, Writing – Review and Editing. Brian Murray – Resources, Conceptualization, Methodology, Writing – Review and Editing. Sridhar Krishnan – Resources, Conceptualization, Methodology, Writing – Review and Editing. Arun N.E. Sundaram – Resources, Writing – Review and Editing. Hans Katzberg - Supervision, Resources, Conceptualization, Methodology, Writing – Review and Editing. Karthikeyan Umapathy – Project administration, Supervision, Resources, Conceptualization, Methodology, Writing – Review and Editing.

## Value of data

The data provide significant value by enabling the development and validation of objective screening methodologies for Myasthenia Gravis, supporting earlier detection and more consistent clinical decision-making. In addition, the dataset validates the developed methods on a real-world patient population affected by the condition, facilitating the potential translation of research findings into scalable, non-invasive screening tools.

## Declaration of competing interest

The authors declare that they have no known competing financial interests or personal relationships that could have appeared to influence the work reported in this paper.

## Data Availability

The data that has been used is confidential.
